# A Simple Coloration of Calcium Alginate Fiber via Structural Colors

**DOI:** 10.3390/polym17212919

**Published:** 2025-10-31

**Authors:** Xinyu Yang, Xing Tian, Yu Zhang, Pengfei Gao, Jianhua Hou, Junyu Zhong

**Affiliations:** 1 State Key Laboratory of Bio-Fibers and Eco-Textiles, College of Materials Science and Engineering, Qingdao University, Qingdao 266071, China; yangxinyu1@qdu.edu.cn (X.Y.); xingtian1982@qdu.edu.cn (X.T.); zhangyu60@qdu.edu.cn (Y.Z.); gaopengfei@qdu.edu.cn (P.G.); 2 College of Environmental Science and Engineering, Yangzhou University, Yangzhou 225009, China; jhhou@yzu.edu.cn

**Keywords:** calcium alginate fiber, silicon dioxide, polydopamine, structural color

## Abstract

Seaweed fiber is a new type of functional fiber made from natural seaweed as raw material. Seaweed fiber has excellent moisture absorption, bio compatibilization, controlled degradation profile, and flame retardancy, and can be used to develop high-performance and high value-added textiles. However, seaweed fibers are prone to swelling in salt ion solutions, making dyeing with traditional chemical dyes very difficult. In recent years, the research and application of controllable structural colors have been an important direction and hot spot in the textile field. SiO_2_ nanospheres of different sizes were synthesized and combined with polydopamine as an additive to produce structural colors with high visibility. The resulting photonic crystals exhibited vibrant rainbow hues and were successfully applied to stain seaweed fibers. The color of polydopamine-coated silica photonic crystals (PDA/SiO_2_) depended on the diameter of the SiO_2_ microspheres, while their spectral purity could be tuned by adjusting the ratio of SiO_2_ microspheres to dopamine hydrochloride.

## 1. Introduction

As an important class of optical materials, structural color materials have shown great application prospects in display devices, sensors, optical wave guides, flexible wearables and so on. However, in most applications, it is necessary to be able to regulate the structural color of the materials, so the research application of tunable structural color has become a particularly important direction and hot spot in the field of structural color textiles [1]. Calcium alginate fiber is a new type of functional fiber produced by a wet spinning process using sodium alginate extracted from natural seaweed as raw material and aqueous calcium chloride solution as coagulation bath. Calcium alginate fiber has excellent moisture absorbency, bio compatibilization, bio-degradability and low environmental impact [2].

Calcium alginate fiber is a natural kelp extract made by wet spinning and is highly hydrophilic and biocompatible. It can be used as a biomedical textile fiber to promote wound healing, and has great prospects for development [3]. Calcium alginate fiber also demonstrates potential for application in the development of high-performance fabrics and decorative textiles suitable for fashion and lingerie manufacturing. However, calcium alginate fiber is easily dissolved in salt ion solution, and chemical pigments are not easily diffused and uniformly dispersed, so it is not easy to apply color, and chemical pigments and dyes usually cause serious environmental pollution during the dyeing process of calcium alginate fiber, and the dyed colors are easily faded over time. In recent years, structured colors generated by the interaction of visible light with photonic nanostructures have attracted worldwide attention due to their high brightness, non-fading and non-polluting properties [1,4,5]. Among these photonic structures, Polydopamine-coated silica photonic crystal (PDA/SiO_2_) has received increasing attention because it can produce angle-dependent rainbow colored structured colors, such as iridescent structured colors, when the structural feature size is comparable to the wavelength of visible light [6,7,8].

This paper presents a new product process for the structural coloring of calcium alginate fiber with high color visibility using polydopamine as an additive, based on a drop-coating process. The photonic crystals (PCs) obtained by self-assembling mixtures of monodisperse silica (SiO_2_) [9] spheres and polydopamine microspheres particles exhibit vivid iridescent structural colors, which can provide a subtle tunable hue that can cover the visible spectrum [10]. PCs are closely related to the diameter of silica microspheres, and PC spectral purity can be regulated by controlling the addition of silica microspheres and dopamine hydrochloride. We went through a simple experimental procedure: the mixture of SiO_2_ microspheres and appropriate amount of dopamine hydrochloride is dropped on calcium alginate spinning material or calcium alginate fabrics and then, dried under specific ambient temperature and humidity, calcium alginate structured color fabrics are obtained. The microstructure and composition of structural PC have been characterized using scanning electron microscopy (SEM), metallurgical optical microscopy, potentiometric particle size analyzer (Zeta), ultraviolet-visible spectrophotometer (UV-Vis) and Fourier-transform infrared spectroscopy (FTIR). The optical properties of the structural PC were investigated using a color-matching system. In addition, the stability of calcium alginate structured color fabrics was investigated using simulated folding experiments.

## 2. Experimental Section

### 2.1. Materials

Reagents and Instruments: Tetraethyl orthosilicate (TEOS, 95%) was purchased from Sinopharm Chemical Reagent Co., Ltd. (Shanghai, China). anhydrous ethanol (EtOH, 99.5%), dopamine hydrochloride (DA, 98%) and hydrochloric (Tris, 99%) acid buffer were purchased from Sinopharm Chemical Reagent Co., Ltd. (Shanghai, China). Ammonia solution (AR, 28–30%) was obtained from Innochem (Beijing, China). Deionized water and calcium alginate fabric were self-prepared in the laboratory. Tetrahydrofuran (THF, 99%) was purchased from Sinopharm Chemical Reagent Co., Ltd. (Shanghai, China).

Instrumentation: JSM-6390 Scanning Electron Microscope (Hitachi, Japan), Data-color 850 color measurement and matching system (Datacolor, Suzhou, China), Model T9 Ultraviolet-Visible Spectrophotometer (Beijing Pulse General Instrument Co., Ltd., Beijing, China), BX53M Metallographic Optical Microscope (Olympus, Tokyo, Japan), Nicolet iS50 infrared spectroscopy (FTIR, Waltham, MA, USA), Zeta potential and particle size Analyser Model 90Plus PALS (Brookhaven, Holtsville, NY, USA).

### 2.2. Synthesis of Monodisperse SiO_2_ Microspheres

Monodisperse SiO_2_ microspheres were prepared by a modified Stöber [11] method. Amounts of 45 mL of anhydrous ethanol and 4 mL TEOS were homogeneously mixed on a stirrer and stirred for 5 min at 1100 r/min and 30 °C; 16 mL of anhydrous ethanol (EtOH), 9.00 mL of ammonia (NH_3_-H_2_O) and 24 mL of deionized water were mixed well and poured into the stirrer for 10 min in turn, and then the speed was adjusted to 400 r/min for 2 h. At the end of the reaction, 10,000 r/min centrifugation was performed for 10 min, and the lower precipitate layer was washed with anhydrous ethanol three times. After the reaction, 10,000 r/min centrifugation was performed for 10 min, and the lower precipitate was centrifuged with anhydrous ethanol at 10,000 r/min three times, dried in an oven at 80 °C for 2 h, then the monodisperse silica microspheres were obtained by grinding named as SiO_2_-1. The remaining products are prepared in the same way as above. The products obtained by changing the temperature conditions of the reaction process to 50 °C and 70 °C are named as SiO_2_-2, SiO_2_-3.

#### 2.2.1. PDA/SiO_2_ Amorphous Photonic Crystal Synthesis

Take 1.00 mL of 1 mol/L hydrochloric acid buffer solution with pH = 8.5 and 100 mL of deionized water, add 1 g of SiO_2_-1 micro-spheres and sonicate the mixture until well dispersed. The mixture was poured into a 50 mL three-necked flask and 0.03 g of DA was added at 500 rpm, 25 °C and the reaction was carried out for 24 h. The product was washed three times with deionized water, filtered and dried in a vacuum oven at 60 °C for 24 h. The amorphous photonic crystals, PDA/SiO_2_-1, were obtained by grinding. PDA/SiO_2_-2 and PDA/SiO_2_-3 photonic crystals were obtained by replacing SiO_2_-1 with SiO_2_-2 and SiO_2_-3, respectively.

#### 2.2.2. Synthesis of Composite Material PDA/SiO_2_/SA

The laboratory prepared calcium alginate spunlace fabric (SA) was taken and pre-treated with tetrahydrofuran by immersion until the fabric was completely wetted. Dissolve 0.5 g of PDA/SiO_2_ powder in 20 mL of deionized water and drop by drop with a dropper to obtain PDA/SiO_2_/SA (calcium alginate fabric). The obtained textured colored seaweed fabric was placed in the oven at 80 °C for 40 min to obtain the textured colored calcium alginate fabric with high brightness.

### 2.3. Structural Characterization and Performance Testing

SEM examination: SEM was used to observe the micro-morphology of the samples and the samples were diluted with deionized water before testing. UV-Vis: The samples were measured by UV-Vis, the powder samples were ground and flattened for the UV-Vis test, and the thin films were tested by UV-Vis spectroscopy; the samples of thin films were 10 mm × 10 mm × 1 mm. Omnidirectional microscope test: The samples were observed by omnidirectional microscope to observe the micro-color and state; the samples were placed flat on the microscope stage before the test. Zeta test: Zeta particle size analysis is used to obtain the average size of the sample. Spiral micrometer test: The thickness of the sample is analyzed using a spiral micrometer to analyze the thickness of different areas of the sample. Color matching system test: A color matching system was used to test the chromaticity and chromatic aberration of the samples; the sample film was placed vertically and aligned with the test holes. The sample size was 10 mm × 10 mm × 1 mm. Spiral micrometer test: The thickness of the sample is analyzed using a spiral micrometer, which analyzes the effect of the thickness of different areas of the sample on the chromaticity of the sample. All photos in this article were taken with a Huawei mate 60 mobile phone.

## 3. Results and Discussion

### 3.1. Characterization of Monodisperse SiO_2_ Microspheres

**Figure 1:**
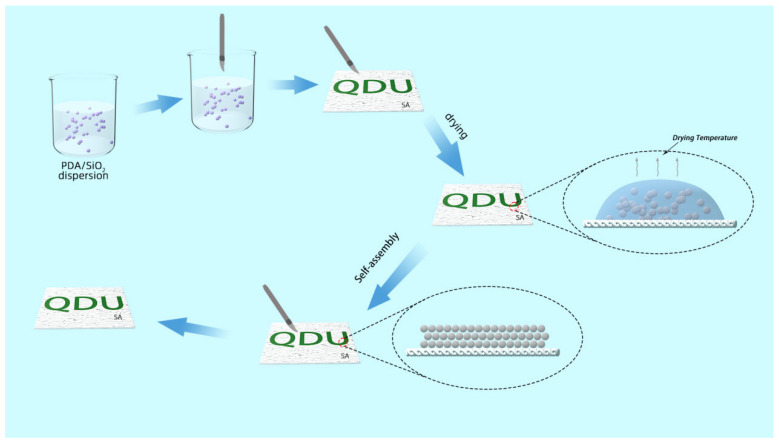
Schematic structure of PDA/SiO_2_/SA fabrication process.

**Figure 2:**
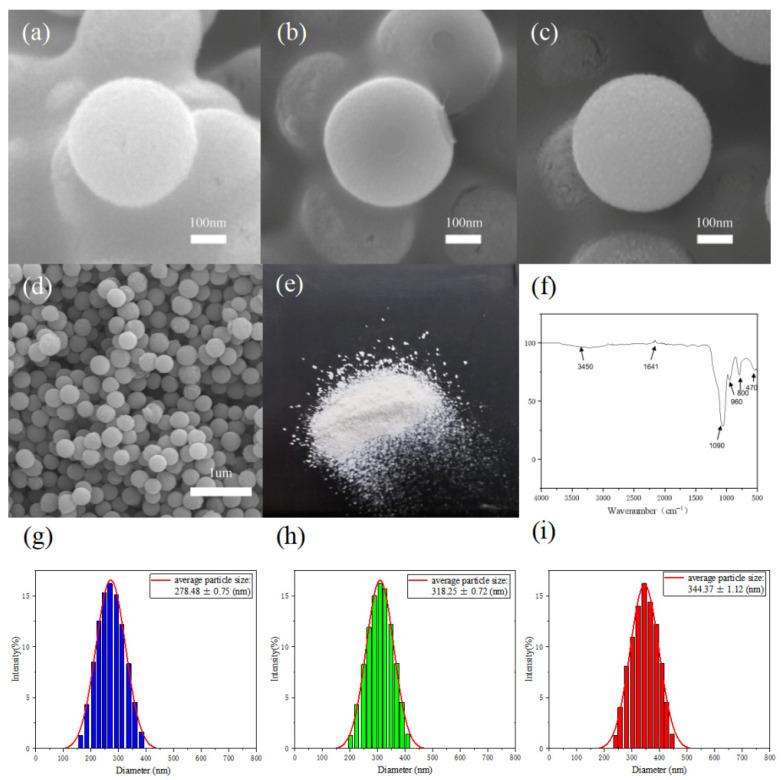
(**a**–**c**) Scanning electron microscope images of monodisperse silica microspheres. (**d**) Scanning electron microscope images of photonic crystals consisting of 278 nm silicon dioxide microspheres mixed with poly-dopamine. (**e**) Optical photographs of silica microspheres at 278 nm. (**f**) FTIR spectrum of SiO_2_. (**g**–**i**) Particle size distribution of monodisperse SiO_2_ microspheres synthesized by controlling the reaction conditions at temperatures of 70 °C, 50 °C and 30 °C.

Figure 1 shows the whole process of structural color dyeing of calcium alginate fiber fabrics. Figure 2a–c shows that uniform and uniformly sized silica microspheres were obtained by controlling the temperature of the reaction process of hydrolysis of tetraethyl orthosilicate. The particle size of the monodisperse SiO_2_ microspheres decreases as the temperature of the reaction process increases, and the average particle sizes of the monodisperse SiO_2_ microspheres. The average particle sizes of the monodisperse SiO_2_-1, SiO_2_-2 and SiO_2_-3 microspheres are 344, 318 and 278 nm, respectively. As shown in Figure 2d, the optical photograph of monodisperse silica powder can be clearly seen; silica powder does not show an obvious optical color. Figure 2e demonstrates that microspheres distribution is very uniform, part of the stacked silica microspheres in the role of gravity in the tightly ordered arrangement, for the formation of a planar cubic structure to provide a very good foundation. Using the Barrg–Snell [12] formula, it can be deduced that the position of the reflection peak formed by the photonic band gap is related to the lattice spacing of the particle size of the monodispersed SiO_2_ microspheres as λ= 2.3874d (where λ is the wavelength of the reflected light, nm, and d is the lattice spacing, nm). The reflection peaks in the visible spectral region (380–780 nm) can be spectrally modulated to match the theoretical values derived from the diameter of the SiO_2_ microspheres [13]. The particle size of monodisperse silica microspheres corresponds to the corresponding theoretical value [14]. Figure 2f shows that the two absorption peaks at 3450 and 1641 cm^−1^ are the antisymmetric stretching vibration peak and bending vibration peak of the -OH bond of water binding in SiO_2_, respectively. The three absorption peaks at 1090, 800 and 470 cm^−1^ are attributed to the antisymmetric telescopic vibration, symmetric telescopic vibration and bending vibration of the Si-O bond, which proves that the monodispersed SiO_2_ microspheres have been successfully prepared [15]. Figure 2g–i depicts the hydrated particle size of monodisperse SiO_2_ microspheres measured by Zeta. The hydrated particle size of the obtained monodisperse SiO_2_ microspheres corresponds to the central wavelength of the photonic band gap as 639, 732 and 762 nm.

### 3.2. Characterization of Monodisperse PDA/SiO_2_ Photonic Crystals

**Figure 3:**
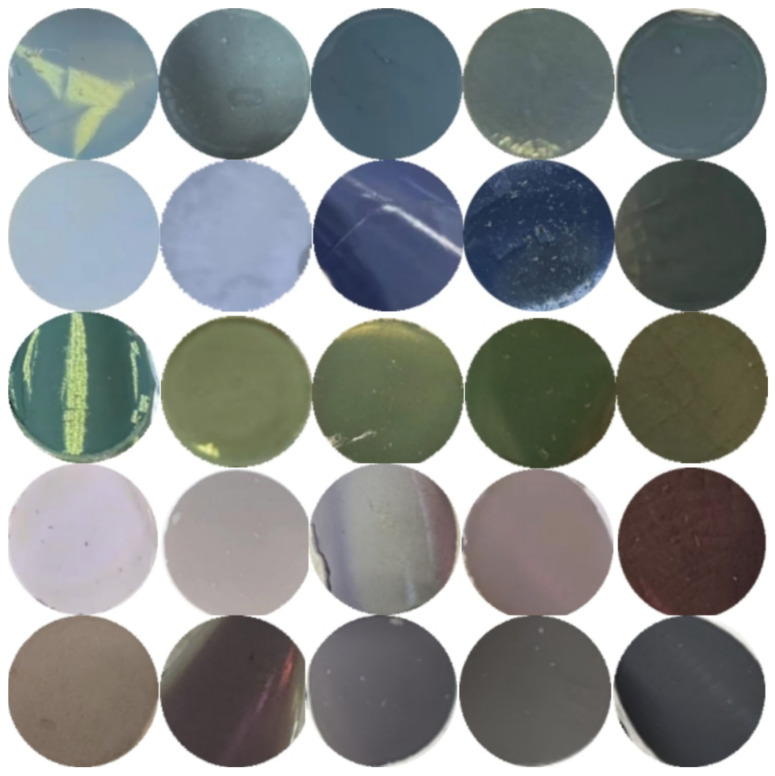
Photonic crystal structure color palette made from PDA/SiO_2_. In general, the diameter of SiO_2_ microspheres in PC decreases from bottom to top, while the proportion of polydopamine increases from left to right.

**Figure 4:**
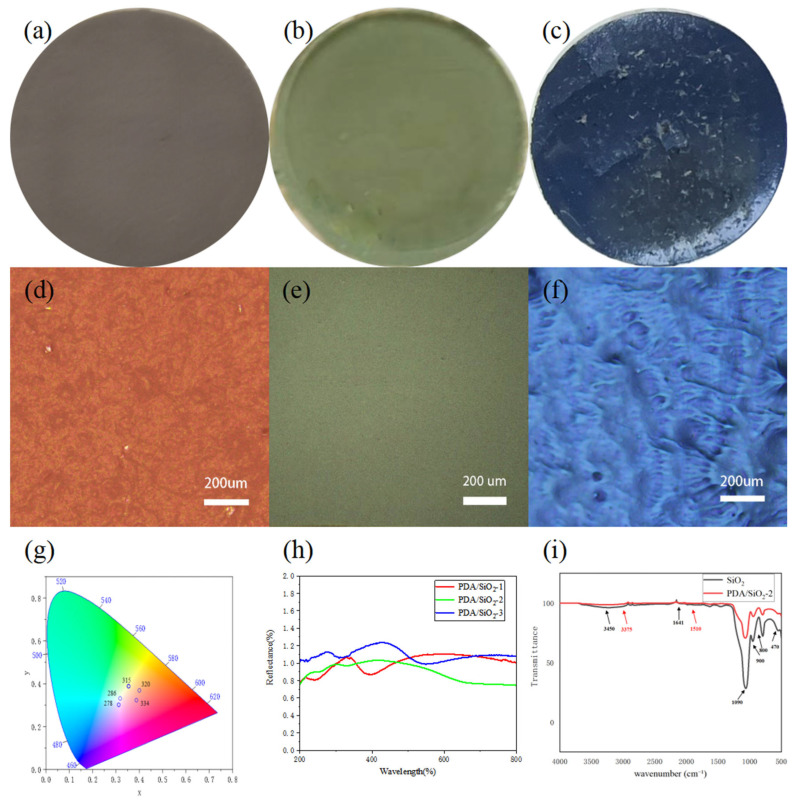
(**a**–**c**) Optical digital photographs of amorphous photonic crystal PDA/SiO_2_ samples, Silicon dioxide particle size 278 nm, 318 nm and 344 nm, respectively. (**d**–**f**) Corresponding micro-optical images in (**a**–**c**). (**g**) CIE chromaticity x and y values for the colors in the palette. Each dot represents photonic crystals with different SiO_2_ microspheres diameters (marked in =) but the same dopamine ratio (increasing from near the white center x = 0.33). Scale bars: (**a**) 5 mm. (**h**) UV-Vis diffuse reflectance spectra of amorphous photonic crystal PDA/SiO_2_. (**i**) FTIR spectrum of PDA/SiO_2_.

In Figure 3, Polydopamine [16] is used as an artificial analogue of melanin, and polydopamine has a similar complex structure and chemical composition to biological melanin. The polymerization of dopamine to polydopamine follows a similar reaction pathway in living organisms as described above, and the polydopamine contains catechol [16,17] groups having superadhesive properties similar to those of mussel foot filaments. Hydrolysis of tetraethyl orthosilicate results in a large number of carboxyl and hydroxyl groups on the surface of the resulting SiO_2_, so that the PDA/SiO_2_ is tightly packed through hydrogen bonding and van der Waals forces, forming a planar cubic structure under gravity to form a highly luminous structural color [7,8,16]. By using SiO_2_ microspheres of different sizes and different ratios of PDA/SiO_2_, PCs [18] of different rainbow colors can be obtained, providing a great palette of structural colors. The design strategy is as follows. The color variation is adjusted by the particle size of the silica microspheres, while the spectral purity is adjusted by the proportion of Polydopamine. Using SiO_2_ microspheres with diameters between 270 and 380 nm, a range of colors from blue to green-red was achieved. As shown in Figure 4d–f, by observing red, green and blue photonic crystal samples under a metallurgical microscope with a large numerical aperture objective, red, green and blue structural colors consistent with reality can still be seen in the microcosm. This unique property of PC is important for certain applications, such as microscopic color displays [11,19,20]. In order to understand how color varies with silica particle diameter and dopamine ratio, we converted the measured PDA/SiO_2_ reflectance spectra into the International Commission on Illumination (CIE) chromaticity values [18] shown in Figure 4g. The measured reflectance spectra were then converted into the CIE chromaticity values. The absorption peak at 3375 cm^−1^ is broadened after SiO_2_-2 is coated with PDA, and the telescopic vibration peaks of hydrogen bonding and -OH bonding between PDA and SiO_2_ overlap, while the bending vibration of N-H bonding appears at 1510 cm^−1^. The absorption peak at 1510 cm^−1^ indicates the successful coating of PDA/SiO_2_-2 in Figure 4i [21].

Images of different PC structures on calcium alginate fiber fabrics are shown in blue, green and red, taken under diffuse light illumination conditions. Since polydopamine has good adhesive properties [2,21], it fills the voids in the SiO_2_ microspheres and aligns the SiO_2_ microspheres tightly together. The sizes of the blue (278 nm SiO_2_ microspheres), green (318 nm SiO_2_ microspheres) and red (344 nm SiO_2_ microspheres) SiO_2_ microspheres correspond to the particle size range of the Bragg–Sneer equation, and the reflectivity of the Bragg stacks is related to the contrast between the refractive indices of the two and the number of layers. As shown in previous studies, the non-rainbow structural colors of PCs are mainly due to coherent scattering caused by short-range order [22]. Therefore, the color of the PC can be adjusted by changing the reflected wavelength. That means different colors can be obtained by using SiO_2_ microspheres of different diameters. An interesting feature of the structural color is that the color varies with the dielectric environment. In fact, a PC composed of 318 nm SiO_2_ microspheres turns from green to dark black after a drop of water. Due to the hydrophilic nature of polydopamine, the response to this color change is quite rapid.

### 3.3. Characterization and Application of PDA/SiO_2_/SA Structured Color Seaweed Fibers

**Figure 5:**
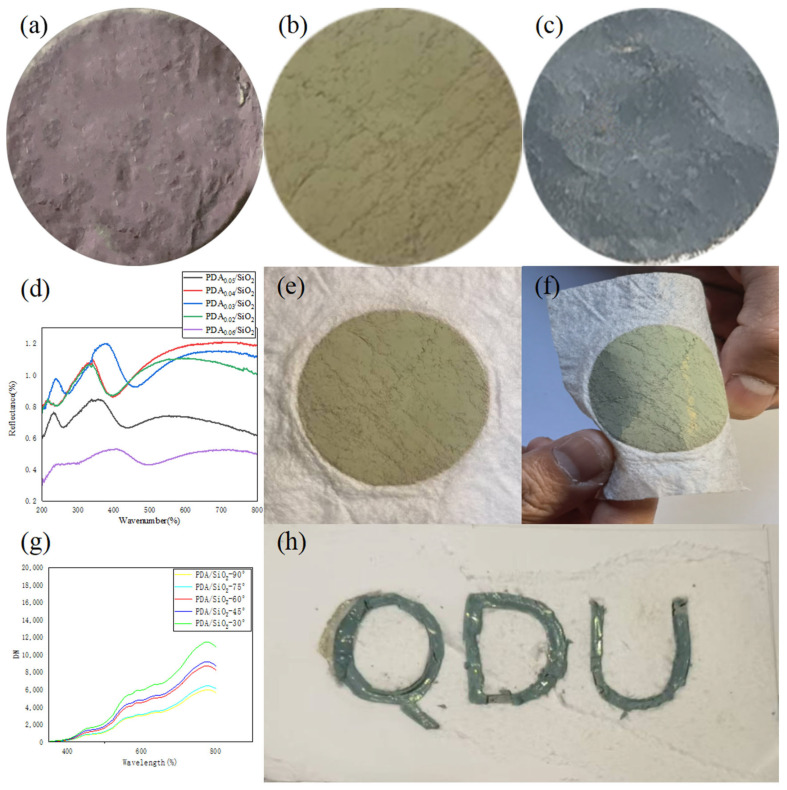
(**a**–**c**) Optical digital photographs of amorphous photonic crystal PDA/SiO_2_ samples, silicon dioxide particle size 278 nm, 318 nm and 344 nm, respectively. (**d**) UV-Vis diffuse reflectance spectra of amorphous photonic crystal PDA/SiO_2_/SA. (**e**,**f**) Optical photographs of PDA/SiO_2_-2/SA. (**g**) Spectral reflectance profile. (**h**) Our university logo is colored in non-rainbow structural colors.

**Figure 6:**
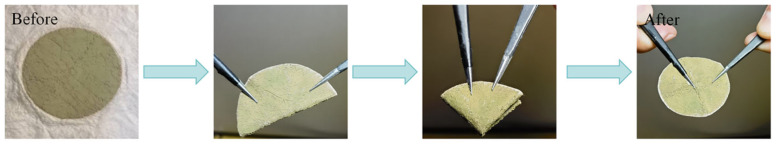
Folding experimental process physical optical picture.

Through the visible spectral graph, we changed the proportion of adding dopamine with the proportion of dopamine increases. The corresponding photonic crystal on the spectrum of the peak decreases, but if the proportion is too low due to the polydopamine broadband, absorption is insufficient to show the SiO_2_ film white. By comparing the optimal ratio of the brightness of photonic crystals, they can be obtained for the SiO_2_:PDA at 30:1 in Figure 5d. As the surface of the calcium alginate spun fabric is relatively smooth and flat, it is conducive to the formation of a planar cubic structure, resulting in blue, green and red colors on the calcium alginate fabric [15,23]. Due to the adhesive property of polydopamine and the large number of amino and hydroxyl groups, the contact area between the SiO_2_ microspheres was increased, resulting in an increase in the bonding strength, and the strength and stability of the structure of PDA/SiO_2_/SA were improved [20,24]. We provided a two-dimensional model of a calcium alginate fabric and a photonic crystal, which are connected by hydrogen bonds and van der Waals forces (Figure S5, Supporting Information). As shown in Figure 5e,f, when we fold and bend the 318 nm PDA/SiO_2_/SA, it can be clearly seen that the green non-rainbow structural color is firmly attached to the surface of the calcium alginate fabric fiber, which proves that the PDA/SiO_2_/SA can be carried flexibly, to increase the color saturation to further improve the structural quality and to obtain a better order of calcium alginate structural color fabrics [12,25]. Therefore, due to the hydrophilicity and high binding strength of PDA, we can tackle the calcium alginate fabrics with tetrahydrofuran [26] in advance during the drop-coating process, which improves the contact angle between the calcium alginate fabrics and PC, resulting in better color saturation. We also found that the wavelength of the visible light reflection did not change no matter how we changed the amount of dopamine; in other words, changing the amount of dopamine did not change the color of the visible light reflection. This means that the color of the structural color is determined by the particle size of the silica. It is interesting to note that during our experiment, the samples reflected the same color with different brightness due to the different angles from which we viewed the same sample in Figure 5g. So, we tested the reflection peaks at different angles using a floor reflect-meter. From Figure 5g, we can clearly see that the peak of reflection is the highest when the angle of physical reflection and calcium alginate structure color fabric is 60 °C. It can be concluded that the sample has the highest brightness when it is 60 °C and at this time, we can observe non-photonic crystal structure color fabric with a rainbow effect. To demonstrate that photonic crystals can be used to make textile patterns on seaweed fabrics [6,19,24,27], we use PCs to create our university logo structured on color fabrics, on calcium alginate fabrics, by the drop-coating method. The green color was achieved using 318 nm SiO_2_ microspheres. The process of exploring the stability of the structural color fabric was carried out by folding the sample PDA/SiO_2_/SA, as can be seen in Figure 6. When we folded the sample once and then folded it again and then opened the fold, we can see that there is a clear crease, and the structural colors are slightly detached. Looking at the shape of PDA/SiO_2_/SA after it was folded three times, we can clearly see the yellow-green color at the crease. It shows that the structural color calcium alginate fabric can resist the external mechanical force, indicating the practicality of structural color calcium alginate fabrics. The color of PDA/SiO_2_/SA is the same as that of the photonic crystal with the same particle size. This is due to the fact that calcium alginate fabrics and photonic crystals are connected by hydrogen bonding and van der Waals force and that no new chemical bonds are created [28].

Calcium alginate fabric has strong water absorption. Before drop coating, the calcium alginate fabric is tackled with tetrahydrofuran, which increases the contact angle between PCs and the seaweed fabric, and makes the seaweed fabric denseness increase further so that PCs are better embodied in the seaweed fabric, and at the same time, it restricts the phenomenon of diffusion of PCs in the seaweed fabric, and is conducive to PCs’ self-assembly on the fabric (Figure S3, Supporting Information).

## 4. Conclusions

In this paper, monodisperse SiO_2_ microspheres with different particle sizes were prepared by controlling the reaction temperature of the hydrolysis process of tetraethyl orthosilicate, and the amorphous photonic crystals PDA/SiO_2_ were produced by using the characteristics of self-polymerization of dopamine hydrochloride under alkaline conditions to coat SiO_2_, and the structured color composites PDA/SiO_2_/SA were prepared by drop-coating on calcium alginate fabrics, and tested by FTIR, SEM, UV-Vis, Zeta, etc. The following conclusions were obtained.

(1) The particle size of monodisperse SiO_2_ microspheres is controllable and homogeneous, and the particle size decreases with the increase of reaction temperature. Three kinds of monodisperse SiO_2_ microspheres with particle sizes of 287, 318 and 344 nm can be produced at reaction temperatures of 70 °C, 50 °C and 30 °C, respectively.
(2) Monodispersed SiO_2_ microspheres with three particle sizes of 278, 318 and 344 nm coated with PDA exhibit blue, green and red amorphous photonic crystals PDA/SiO_2_.
(3) With the successful introduction of the amorphous photonic crystal PDA/SiO_2_ into SA, a structure-colored composite material, PDA/SiO_2_/SA, has been produced.

In this paper, the preparation of structural color materials ensures a streamlined process while strengthening the solidity within the photonic crystal structure, resulting in a more stable structural color. It provides a research basis for further solving the problems of calcium alginate fabrics that are not easy to be colored and are easy to fade, and provides ideas for the subsequent preparation of richer seaweed fabric dyeing applications.

## Data Availability

The original contributions presented in the study are included in the article/Supplementary Materials; further inquiries can be directed to the corresponding authors.

## Supplementary Materials

The following supporting information can be downloaded at https://www.mdpi.com/article/10.3390/polym17212919/s1, Figure S1: (a) SEM photographs of PDA/SiO_2_ structured colour photonic crystals. (b) SEM photographs of SiO_2_ aggregated states. (c) SEM photographs of SiO_2_ aggregation states after PDA capping. (d) SEM photographs of structured colour photonic crystals attached to seaweed fiber; Figure S2: Values of K/S absorbed by different structural colour photonic crystals; Figure S3: (a) Calcium alginate fabric contact angle test with water. (b) Contact angle test of calcium alginate fabric with photonic crystal solution. (c) Contact angle test of tetrahydrofuran pretreated calcium alginate fabrics with photonic crystal solutions; Figure S4: Optical photographs of calcium alginate fabrics. Figure S5: Two-dimensional modeling of calcium alginate fabrics and photonic crystals.
