# Supplementary material for: A Simple Coloration of Calcium Alginate Fiber via Structural Colors
Source: Polymers (Basel). 2025 Oct 31;17(21):2919. doi: 10.3390/polym17212919 (PMC12610765; doi:10.3390/polym17212919)
Supplement: Supplementary file 1 [file polymers-17-02919-s001.zip › polymers-3589894-supplementary.pdf]

Article

Study on structural color staining of seaweed fibers

Xinyu Yang[1], Xing Tian[1], Yu Zhang[1], Pengfei Gao[1], Jianhua Hou[2], Junyu Zhong[1*]

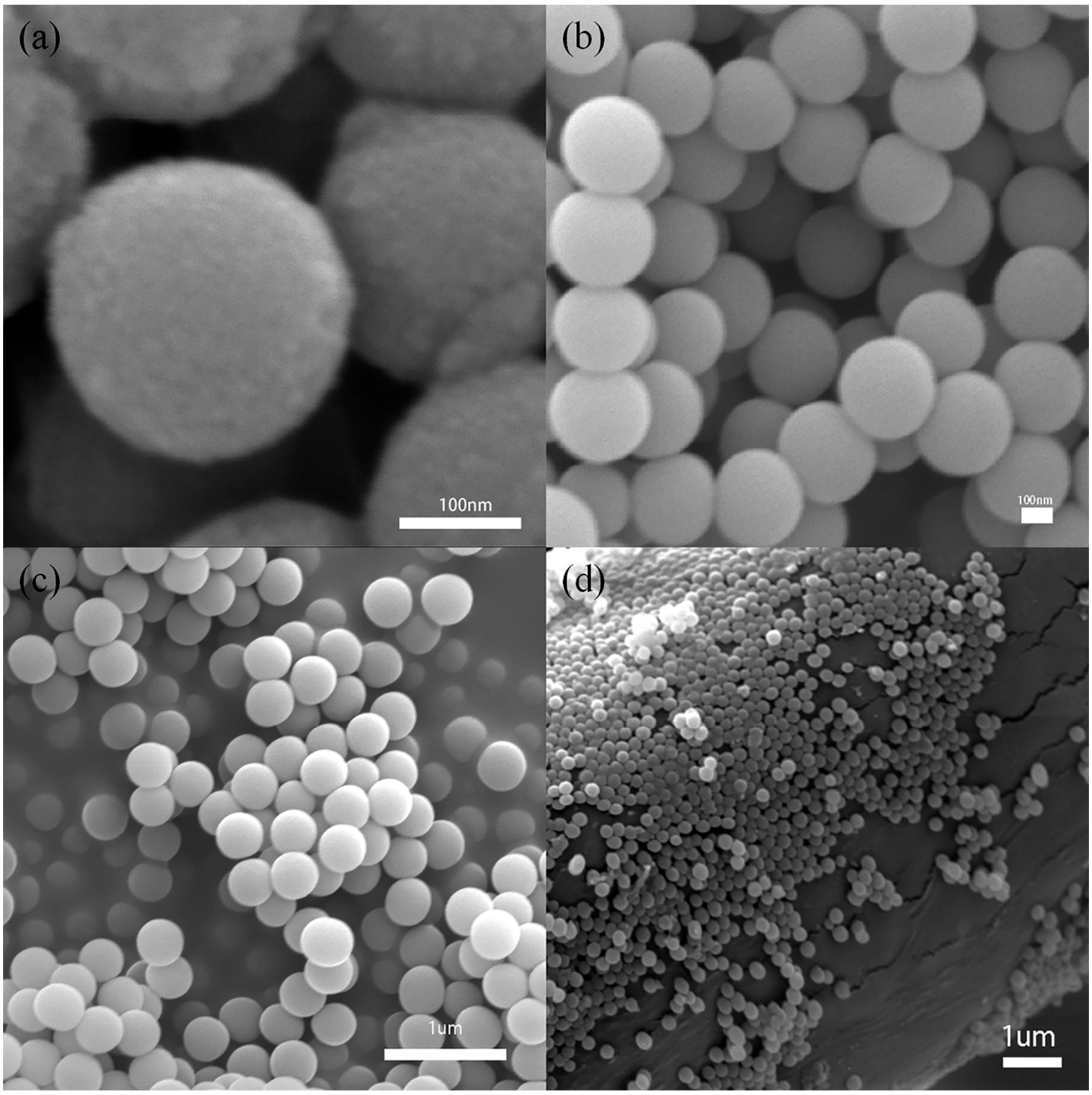

Figure S1 (a) SEM photographs of PDA/SiO_2 structured colour photonic crystals . (b)SEM photographs of SiO_2 aggregated states . (c)SEM photographs of SiO_2 aggregation states after PDA capping . (d)SEM photographs of structured colour photonic crystals attached to seaweed fiber

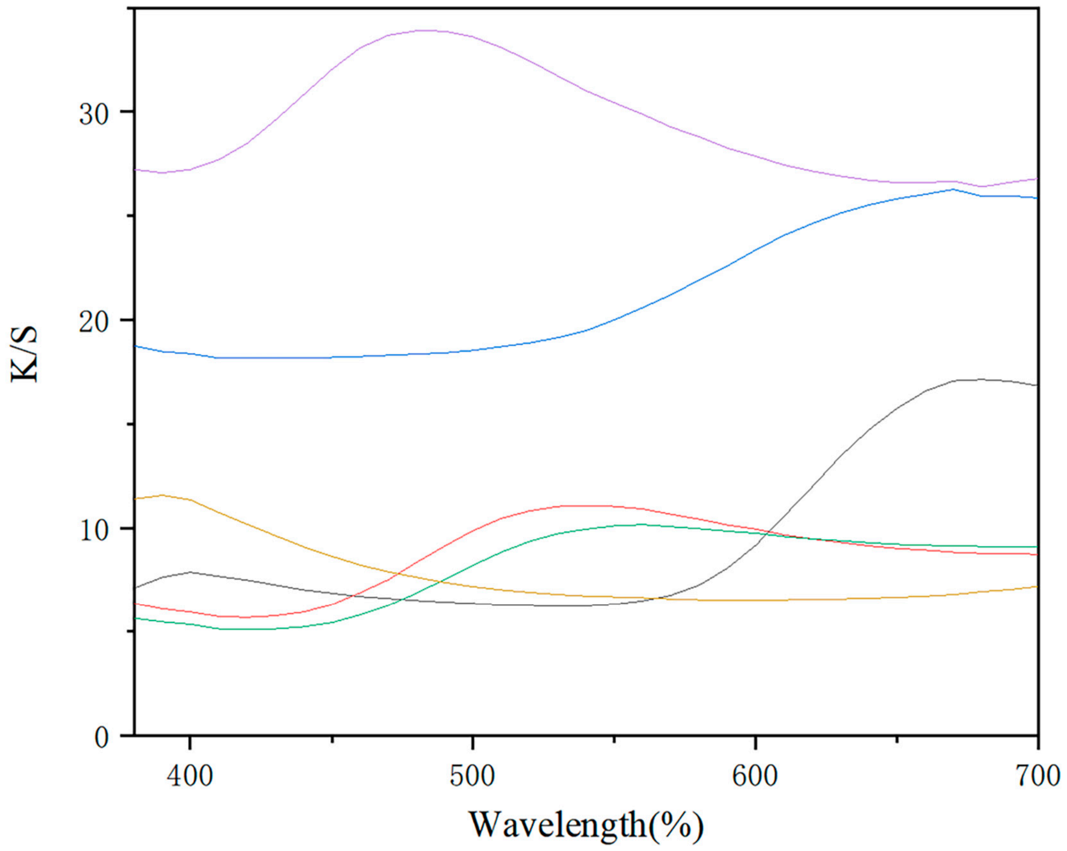

Figure S2 Values of K/S absorbed by different structural colour photonic crystals

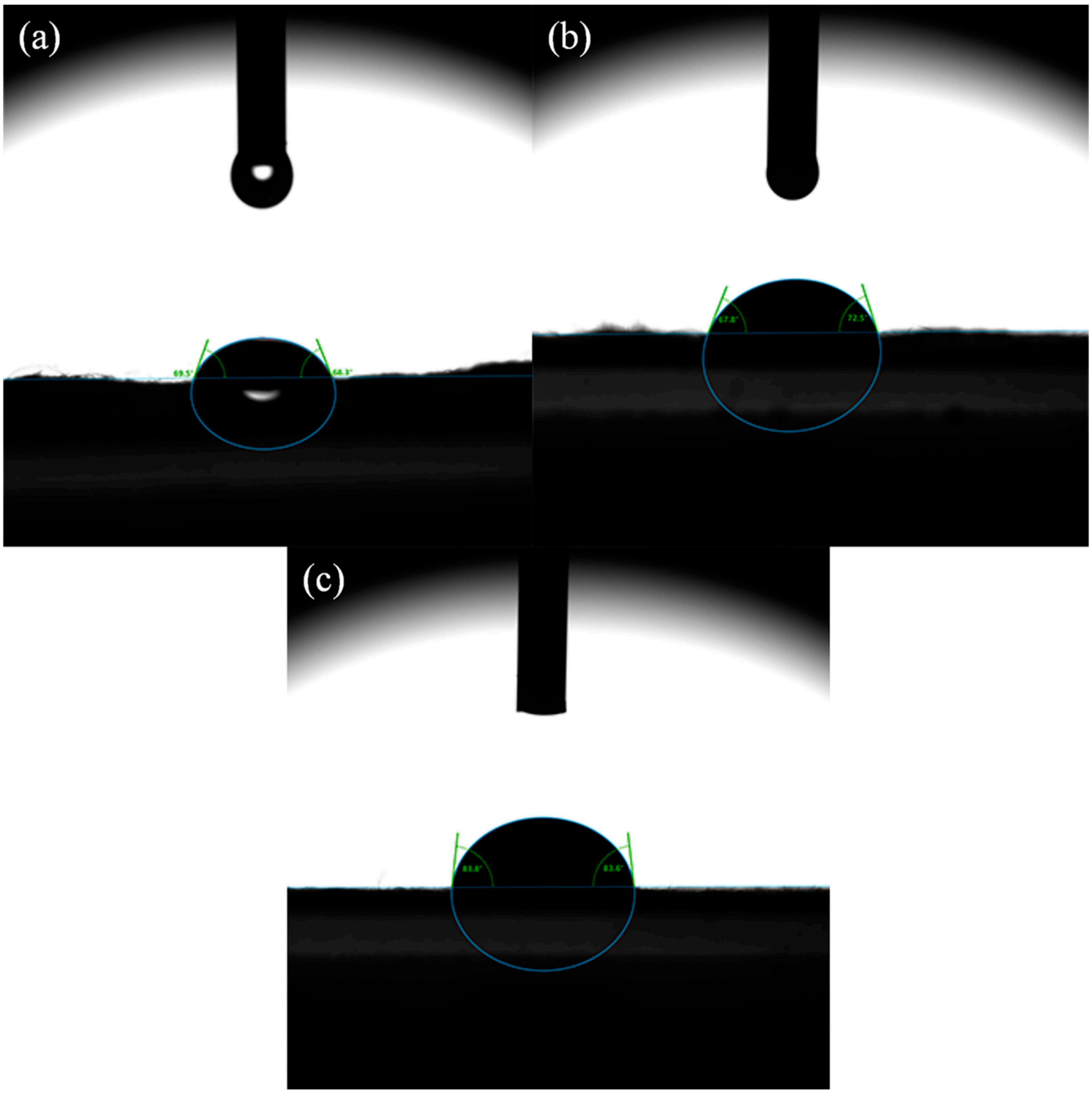

Figure S3 (a) Calcium alginate fabric contact angle test with water . (b)Contact angle test of calcium alginate fabric with photonic crystal solution . (c)Contact angle test of tetrahydrofuran pretreated calcium alginate fabrics with photonic crystal solutions .

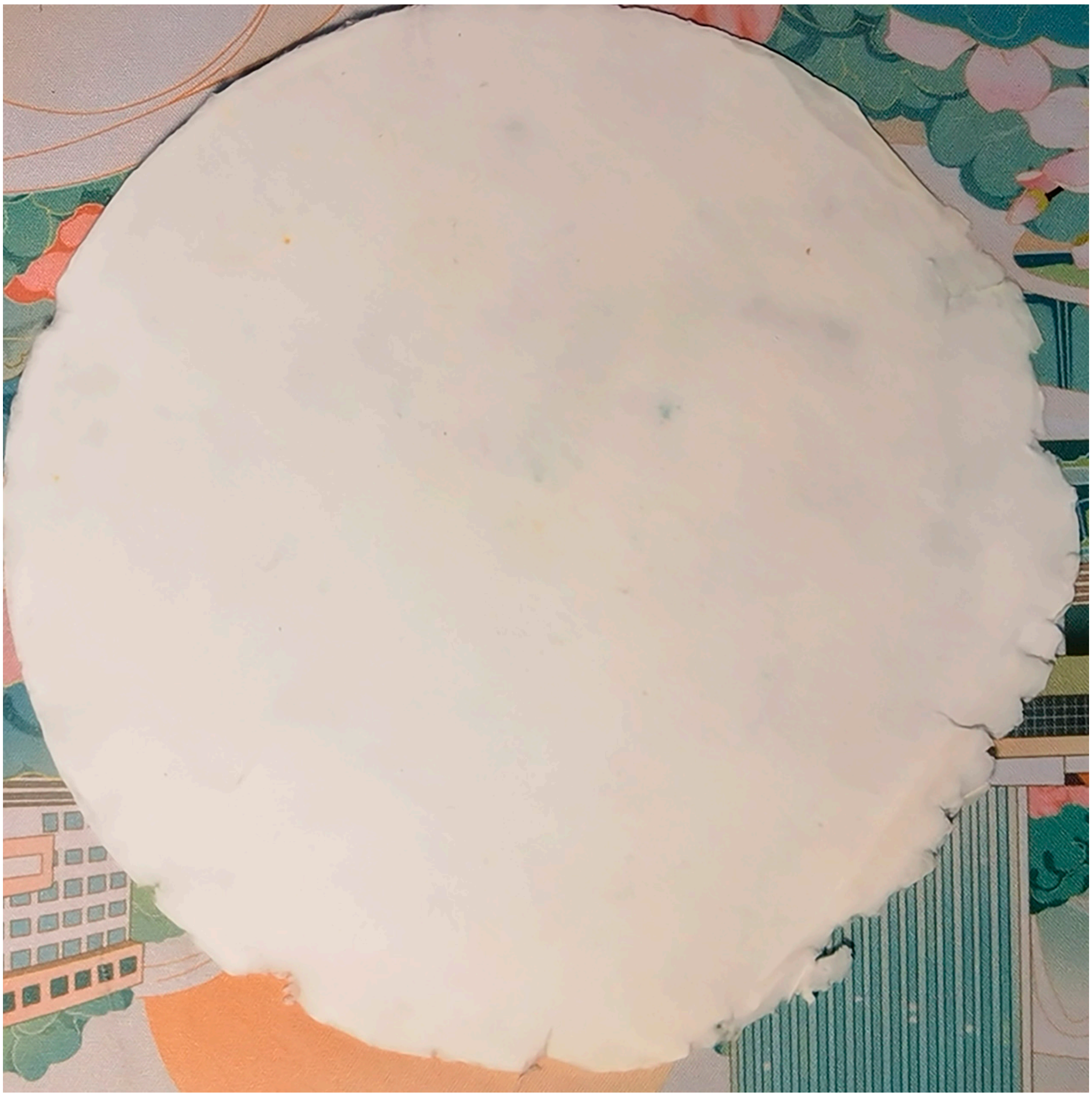

Figure S4 Optical photographs of calcium alginate fabrics .

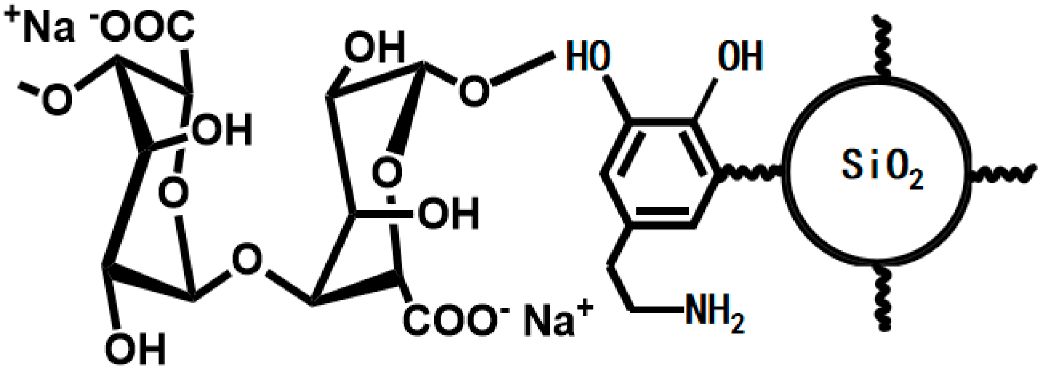

Figure S5 Two-dimensional modeling of calcium alginate fabrics and photonic crystals.
